# Farmacogenética del cáncer colorrectal en un hospital terciario de Valencia

**DOI:** 10.1515/almed-2024-0063

**Published:** 2024-09-24

**Authors:** Ana Comes-Raga, Luis Sendra, Goitzane Marcaida-Benito, Salvador F. Aliño, María José Herrero

**Affiliations:** Servicio de Análisis Clínicos, Consorcio Hospital General Universitario de Valencia, Valencia, España; Departamento de Farmacología, Universitat de Valencia y Plataforma de Farmacogenética, IIS La Fe, Valencia, España

**Keywords:** farmacogenética, medicina de precisión, cáncer colorrectal, polimorfismo, variante, genotipado

## Abstract

**Objetivos:**

Las variaciones genéticas que afectan a procesos farmacocinéticos y farmacodinámicos influyen en la aparición de reacciones adversas y supervivencia de pacientes en tratamiento de cáncer colorrectal.

**Métodos:**

Se realizó una selección de variantes genéticas según la quimioterapia pautada junto con las bases de datos farmacogenéticas. El genotipado se realizó con la tecnología MassArray (Agena Bioscience). Se realizaron estudios de asociación entre variantes-toxicidad y supervivencia-genotipo con métodos de regresión logística (SPSS ver. 28.0.1.1).

**Resultados:**

Se realizó el genotipado de 25 SNPs en 96 pacientes. Para el gen *DPYD*, un 3,5 % presentaron la mutación rs75017182, 4,7 % rs1801158 y 7,1 % rs1801160. Las frecuencias genotípicas en el gen *UGT1A1* fueron 39,4 % (*1/*1), 37,9 % (*1/*28), 19,7 % (*28/*28), y 3 % (*1/*36).

Los genotipos CT de la variante rs1801160, AT de la variante rs67376798 (*DPYD*), y *1/*36 (*UGT1A1*), se relacionaron con eventos de menor supervivencia (p-valor: 0.006, <0.001, y 0.052, respectivamente). La reacción adversa más frecuente fue la gastrointestinal, seguida de neurotoxicidad. El genotipo CC (rs1801160, *DPYD*) se asoció con un menor riesgo de desarrollar toxicidad gastrointestinal grave, y CC (rs1801158, *DPYD*) con un menor riesgo de desarrollar toxicidad hematológica general y grave.

**Conclusiones:**

Nuestro estudio ha puesto de manifiesto que existen diferencias en las frecuencias poblacionales de nuestra serie de pacientes para el rs1801160 y rs75017182 (*DPYD*), *1/*28, *28/*28 y *1/*36 (*UGT1A1*) y las descritas en población española. Se asoció una menor supervivencia del genotipo CT de rs1801160, el genotipo AT de la variante rs67376798 (*DPYD*), y 1/*36 (*UGT1A1*).

## Introducción

La farmacogenética estudia la relación existente entre el impacto de la constitución genética sobre la respuesta y el desenlace clínico de un medicamento en cada paciente. La eficacia y toxicidad a un determinado fármaco está influenciada por la variación genética de cada paciente [1].

Los agentes quimioterápicos en general presentan un estrecho margen terapéutico, por lo que la variabilidad interindividual en su metabolismo determina tanto su eficacia como su seguridad. Para el tratamiento del cáncer colorrectal (CCR) se emplea un tratamiento quimioterápico muy variado que puede dar lugar a múltiples combinaciones de fármacos, entre las que destacamos las fluoropirimidinas como base del tratamiento. Éstas se suelen administrar en combinación con compuestos de platino o con irinotecán [2].

Las fluoropirimidinas pueden causar reacciones adversas graves como son mucositis, diarrea y mielosupresión, incluso pueden provocar la muerte en hasta el 1 % de los pacientes. Esta toxicidad a menudo es causada por la actividad reducida de la enzima dihidropirimidina deshidrogenasa (DPD), la principal enzima metabólica para la inactivación de la fluoropirimidina, la cual está codificada por el gen *DPYD.* Existen distintas variantes de este gen que pueden disminuir la actividad de esta enzima, provocándose los efectos adversos antes descritos [3].

Los compuestos de platino (oxaliplatino y cisplatino) se utilizan en combinación con las fluoropirimidinas para el tratamiento del CCR, cuyo uso tampoco está exento de reacciones adversas [4].

SN-38 es el metabolito activo del irinotecan, y actúa bloqueando la acción de la topoisomerasa I, lo que afecta la replicación y la transcripción del ADN. El gen *UGT1A1* media la destoxificación y conjugación del componente activo SN-38, por lo que variaciones en el mismo pueden provocar reacciones adversas tales como neutropenia, mielosupresión y diarrea en pacientes sometidos a este tratamiento [5].

Plataformas como PharmGKB, el Clinical Pharmacogenetics Implementation Consortium (CPIC) y Dutch Pharmacogenetics Working Group realizan revisiones periódicas de la literatura existente y elaboran guías de aplicación clínica de la farmacogenética, lo que ha permitido que se conozcan los genes asociados con procesos farmacocinéticos y genes asociados a mecanismos farmacodinámicos [6, 7]. También las agencias reguladoras de medicamentos, tanto la Food and Drug Administration (FDA) como la Agencia Europea de Medicamentos (EMA), incluyen información farmacogenética en las fichas técnicas de los medicamentos empleados en el tratamiento del CCR [8, 9].

A nivel nacional, la Sociedad Española de Farmacogenética y Farmacogenómica (SEFF) y la Sociedad Española de Oncología Médica (SEOM) publicaron una guía clínica para el genotipado de 6 variantes en el gen *DPYD* en pacientes candidatos a tratamiento con fluoropirimidinas [10].

En este estudio nos propusimos realizar el genotipado de las variantes genéticas relacionadas con los farmacogenes de la terapia antineoplásica del CCR en un conjunto de pacientes diagnosticados de este cáncer en nuestro centro, con el objetivo de conocer la presencia de estas variantes en nuestra población, e identificar biomarcadores genéticos asociados a efectos adversos y/o disminución de eficacia.

## Materiales y métodos

Se realizó un estudio observacional retrospectivo en el que se analizaron las variantes genéticas implicadas en metabolismo de fármacos antineoplásicos empelados para el tratamiento del CCR, en muestras de pacientes diagnosticados de esta neoplasia en nuestro centro (Consorcio Hospital General Universitario de Valencia, CHGUV). Para ello, se realizó una búsqueda de las variantes involucradas, en las publicaciones y recomendaciones de las organizaciones anteriormente citadas. Se seleccionaron un total de 25 variantes genéticas. Los genes y sus variantes polimórficas se detallan en la Tabla 1.

**Tabla 1:** Genes y variantes relacionadas con la farmacogenética del CCR analizadas en el proyecto de investigación.

Genes	Variantes	Nivel de EVIDENCIA^a^
*DPYD*	rs3918290	1 A
	rs67376798	1 A
	rs55886062	1 A
	rs75017182	1 A
	rs115232898	1 A
	rs1801158	1 A
	rs1801160	1 A
	rs1801266	1 A
	rs1801268	1 A
	rs59086055	1 A
	rs777425216	NA
	rs78060119	1 A
	rs80081766	NA
*GSTP*	rs1695	3
*XPC*	rs2228001	3
*ERCC1*	rs3212986	3
	rs11615	3
*XRCC1*	rs25487	2 B
*UGT1A1*	*1/*1	1
	*28/*28	1
	*1/*28	1
	*1/*36	1
	rs4148323	1 B
*SEMAC*	rs7779029	3
*C8orf34*	rs1517114	3

^a^Según la base de datos Pharmgkb.

Los pacientes que participaron en el estudio fueron aquellos mayores de edad diagnosticados de CCR en nuestro centro, sometidos a tratamientos oncoterapéuticos y que completaron un año de tratamiento y seguimiento; mientras que aquellos que no se sometieron a tratamientos oncoterapéuticos, o bien rechazaron su participación, fueron excluidos.

Las variables de estudio fueron las relacionadas con la demografía (edad al diagnóstico, sexo, fecha de nacimiento y etnia), datos del tumor (fecha de diagnóstico, estadio del tumor, presencia/ausencia de metástasis), esquema de tratamiento empleado, reacciones adversas (RAMs), y evaluación de la respuesta. Todos los datos se recopilaron a la fecha del diagnóstico, a excepción de las RAMs y la evaluación de la respuesta que se recogieron al año de diagnóstico.

Para la clasificación de las RAMs se empleó la Common Toxicity Criteria of Adverse Events (CTCAE v 4.03) [11], y los tipos de RAMs registrados fueron: neurotoxicidad, toxicidad gastrointestinal (GI), hematológica, renal, cutánea y hepática, y la gravedad de cada una (leve: grado 1 y 2; grave≥3).

Para la evaluación de la enfermedad, los oncólogos se basaron en los Criterios de Evaluación de Respuesta en Tumores Sólidos (RECIST v1.1) [12].

El proyecto fue aprobado por el Comité Ético de nuestro centro. Las muestras recolectadas y analizadas fueron ADN aislado a partir de sangre total obtenida por venopunción. Los SNPs fueron analizados en la plataforma MassArray (Agena Bioscience).

Los resultados se analizaron con el software SPSS ver. 28.0.1.1. Para los estudios de asociación entre los SNPs y las toxicidades se empelaron métodos estadísticos basados en regresión logística, significación de p-valor y expresión de odds ratio. Para los estudios de asociación entre supervivencia y los genotipos expresados por la población, se emplearon regresiones logísticas para variables binarias, con expresión en gráficas de Kaplan–Meier. El evento de supervivencia fue evaluado al año del diagnóstico de la neoplasia en cada paciente.

## Resultados

El estudio reclutó un total de 96 sujetos, 39 % mujeres y 56 % hombres, con edades comprendidas entre 28–83 años, y una mediana de edad de 67 años. Todos los pacientes tenían nacionalidad española, a excepción de 1 paciente norteamericano, 3 pacientes sudamericanos, 1 paciente ruso y 1 paciente búlgaro.

**Figura 1:**
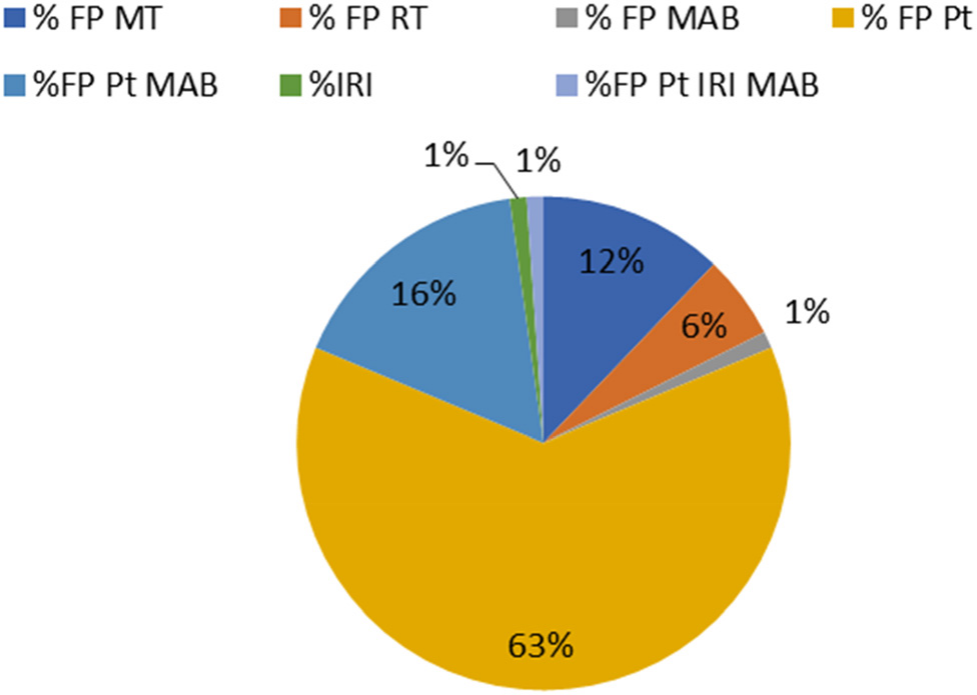
La imagen muestra un diagrama de los esquemas terapéuticos empleados en primera línea de tratamiento para los pacientes del estudio. MAB (anticuerpos monoclonales): bevacizumab, y panitumumab; compuestos de Pt (compuestos de platino): cisplatino, oxaliplatino; FP (fluoropirimidinas): 5-fluorouracilo, capecitabina; IRI (irinotecan).; RT (radioterapia); MT (monoterapia).

Al diagnóstico el 6 % de los pacientes presentaron un estadio I, el 21 % estadio II, el 29 % estadio III, y el 37 % estadio IV. El 20,4 % de los pacientes presentaron hallazgos compatibles con metástasis. Los esquemas terapéuticos empleados fueron fluoropirimidinas (5-fluorouracilo o capecitabina) tanto en monoterapia o junto con sesiones de radioterapia, así como combinados con compuestos de platino (cisplatino u oxaliplatino), y con adición de anticuerpos monoclonales (bevacizumab o panitumumab), e irinotecan. Las distintas combinaciones se muestran en la Figura 1. El 34 % de los pacientes del estudio recibió una segunda línea de tratamiento, y el 6,6 % hasta 3 líneas de tratamiento para su neoplasia.

Se estudió un total de 25 SNPs. Los resultados se muestran en la Tabla 2, donde se recogen todos los resultados de las frecuencias obtenidas, según genotipo salvaje o wild type (WT), homocigoto mutado (HomMut) y heterocigoto mutado (HetMut).

**Tabla 2:** Resultados de los estudios de genotipado de nuestra población.

	Gen *DPYD*														
%	rs3918290 (*2)	rs67376798-DPYD*14	Rs55886062 (*13)	rs75017182 G>C	rs56038477	rs115232898	rs1801158	rs1801160	rs1801266	rs1801268	rs59086055	rs777425216	rs78060119	rs80081766	rs9996584
Wild type	100	99	100	96.5	96.5	100	95.3	92.9	100	100	100	100	100	100	31.8
HetMut	0	1	0	3.5	3.5	0	4.7	7.1	0	0	0	0	0	0	51.8
HomMut	0	0	0	0	0	0	0	0	0	0	0	0	0	0	16.4

	Gen *GSTP*	Gen *XPC*	Gen *ERCC1*	Gen *XRCC1*	Gen *ERCC1*	Gen *SEMAC*	Gen *C8orf34*	*UGT1A1*		
	rs1695	rs2228001	rs3212986	rs25487	rs11615	rs7779029	rs1517114	rs4148323	*1/*1	39.4
Wild type	8.2	30.6	2.4	41.2	9.4	1.2	14.1	100	*28/*28	19.7
HetMut	34.1	54.1	37.6	44.7	45.9	14.1	41.2	0	*1/*28	37.9
HomMut	56.5	15.3	60.0	41.2	44.7	84.7	44.7	0	*1/*36	3.0

Los estudios se expresan en % de pacientes que expresan el genotipo salvaje (wild type), heterocigoto mutado (HetMut) y homocigoto mutado (HomMut). De modo independiente se muestran los resultados para el gen *UGT1A1*.

Para el caso del gen *DPYD*, se puede apreciar que se ha identificado en un 3,5 % de pacientes HetMut para la variante rs75017182, un 4,7 % para la variante rs1801158, y un 7,1 % para la variante rs1801160.

Por lo que respecta al estudio del gen *UGT1A1*, un 77,3 % de los pacientes presentaron un genotipo *1/*1 y *1/*28, un 19,7 % un genotipo *28/*28, un 3 % fueron *1/*36; y el 100 % de los pacientes presentaron un genotipo WT para la variante rs4148323.

Para la variante rs7779029 del gen *SEMAC*, un 84,7 % de los pacientes presentaron un genotipo HomMut, el 14,1 % HetMut y un 1,2 % el genotipo WT; y para la variante rs1517114 del gen *C8orf34* el 14,1 % presentó un genotipo WT, un 41,2 % HetMut y el 44,7 % HomMut.

En cuanto a los compuestos de platino, se estudiaron las variantes rs1695 del gen *GSTP* con un 8,2 % de WT, un 34,1 % de HetMut, y un 56,5 % de HomMut; la variante rs2228001 del gen *XPC* (30,6 % WT, 54,1 % HetMut, y 15,3 % HomMut); la variante rs3212986 del gen *ERCC1* (2,4 % WT, 37,6 % HetMut, y 60 % HomMut); la variante rs25487 del gen *XRCC1* (41,2 % WT, 44,7 % HetMut, y 41,2 % HomMut); y la variante rs11615 del gen *ERCC1* (9,4 % WT, 45,9 % HetMut, y 44,7 % HomMut). Todos estos datos también se muestran en la Tabla 2.

**Figura 2:**
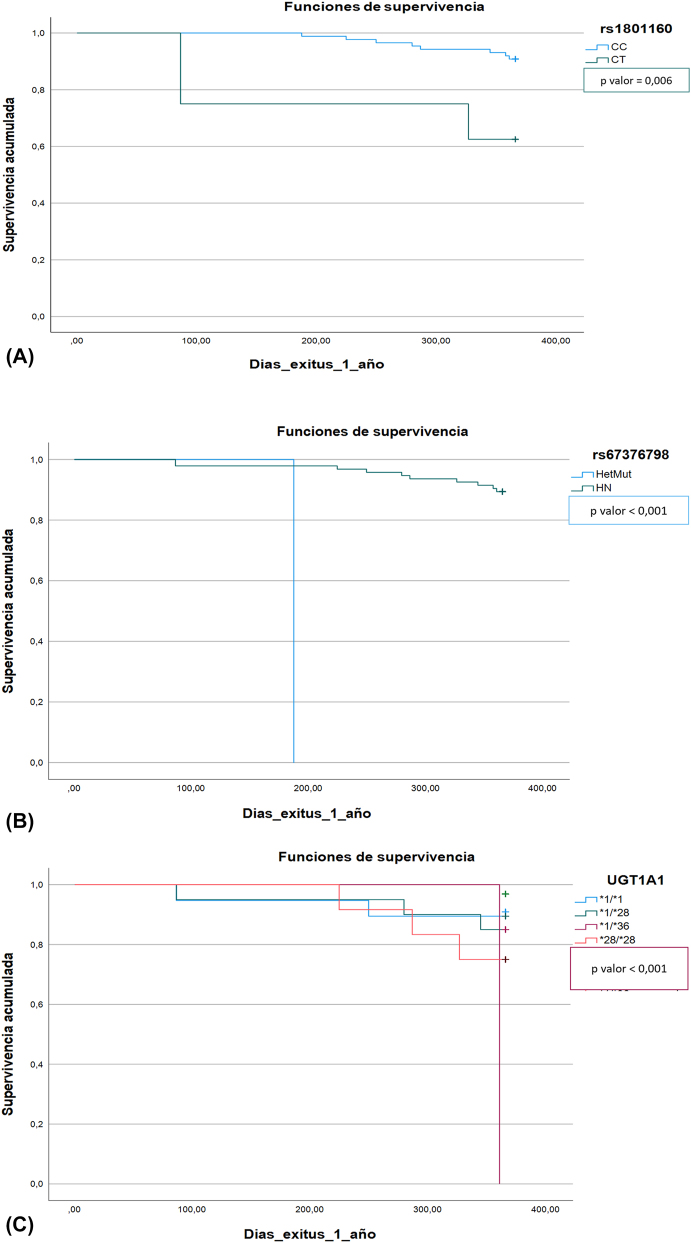
Representación de los resultados obtenidos en gráficas de supervivencia de Kaplan–Meier. La de la izquierda. (A) corresponde a la variante rs1801160 (genotipos CC, CT); al centro (B) la gráfica correspondiente a la variante rs67376798 y el genotipo AT (heterocigoto mutado) y wild type (TT); a la derecha. (C) se representa la gráfica del gen *UGT1A*1 y sus distintas variantes estudiadas.

En los resultados de supervivencia de los SNPs estudiados, los genotipos CT de la variante rs1801160 del gen *DPYD*, AT (Heterocigoto mutado) de la variante rs67376798 del gen *DPYD*, y la variante *1/*36 del gen *UGT1A1*, presentaron niveles de significación de 0.006, <0,001, y 0,052 respectivamente, con eventos de menor supervivencia. Las curvas de supervivencia se muestran en la Figura 2.

El estudio de las RAMs proporcionó los siguientes resultados:

– Neurotoxicidad: un 42 % de los pacientes presentaron neurotoxicidad, siendo RAMs categorizadas como leves en un 40,6 % y graves en un 59,3 %.
– Gastrointestinal, gastrointestinal: se presentó en un 68 % de nuestros pacientes, y en el 49 % de los casos fue de tipo leve y en un 51 % grave.
– Hematológica: fue presentada por un 32 % de nuestra población, siendo en su mayoría (75 %) de tipo grave.

La relación entre el tipo de tratamiento oncoterapéutico y la variedad de RAMs presentadas se expresa en la Tabla 3. En ella podemos observar que todos los pacientes presentaron más de un tipo de RAM durante la administración de su tratamiento. Las RAMs de tipo gastrointestinal predominaron en los esquemas terapéuticos basados en fluoropirimidinas y radioterapia, fluoropirimidinas y compuestos de platino, y fluoropirimidinas junto con compuestos de platino, irinotecan y anticuerpos monoclonales asociados. También estuvo presente en un mismo porcentaje que las RAMs hematológicas en los esquemas basados en fluoropirimidinas con anticuerpos monoclonales asociados, y con igual porcentaje que las RAMs neurológicas en el caso de tratamiento con irinotecan.

**Tabla 3:** Frecuencia de RAMs según el esquema terapéutico administrado.

Esquema terapéutico	% RAM Hematológica		% RAM Gastrointestinal		% RAM Neurológica	
	Leve	Grave	Leve	Grave	Leve	Grave
Fluoropirimidinas (monoterapia)	7.7	NA	38.5	7.7	46.2	NA
Fluoropirimidinas + radioterapia	14.3	NA	28.6	42.9	14.3	NA
Fluoropirimidinas + MAB	50.0	NA	NA	50.0	NA	NA
Fluoropirimidinas + C.Platino	48.8	2.4	58.5	22.0	19.5	7.3
Fluoropirimidinas + C.Platino + MAB	46.2	3.8	46.2	19.2	23.1	7.7
Fluoropirimidinas + C.Platino + MAB + irinotecan	NA	NA	100.0	NA	NA	NA
Irinotecan	NA	NA	NA	50.0	NA	50.0

NA, no aplica; MAB, anticuerpos monoclonales; C.Platino, compuestos de platino.

Se encontraron asociaciones entre el genotipo CC de la variante rs1801160 del gen *DPYD* con un menor riesgo de desarrollar toxicidad GI grave (p valor<0,001; odds ratio 0,064). Del mismo modo, el genotipo CC de la variante rs1801158 del gen *DPYD* también presentó un menor riesgo de desarrollar toxicidad hematológica, en general (p-valor<0,087; odds ratio 0,133) y grave (p-valor < 0,001; odds ratio 0,058). Esta variante se clasificó como protectora frente a eventos adversos graves hematológicos.

No se encontraron asociaciones entre los genotipos estudiados y las toxicidades neurológica, renal, cutánea y hepática.

## Discusión

Los resultados del presente estudio indican que alrededor de un 15 % de los pacientes diagnosticados de CCR presentan alguna variante del gen *DPYD* asociada a pérdida de función de la enzima DPD, con el consecuente incremento de la toxicidad relacionada con el tratamiento con fluoropirimidinas. Estos resultados son más elevados que los que se describen en otros estudios en población española, cuyos resultados son de alrededor de un 10 % [13].

La variante más frecuente es la rs1801160, la variante más frecuente es la rs1801160 (6 pacientes), seguida de la rs75017182 (5 pacientes) y la variante rs1801158 (4 pacientes).

La variante rs1801160 presenta una frecuencia poblacional del 5,6 % para el genotipo CT y un 1,9 % para el TT, en población Caucásica según la literatura consultada. En nuestra serie, el genotipo CT muestra una frecuencia del 7,1 % y no se ha descrito ningún caso homocigoto mutado (TT).

La variante rs75017182 se encuentra en desequilibrio de ligamiento con rs56038477, ambas vinculadas al haplotipo HapB3, y presenta una frecuencia poblacional de 3,7 % según las fuentes de datos para población española. En nuestra serie esta frecuencia se ha descrito en un 3,5 %. Estas dos variantes no siempre se encuentran en desequilibrio de ligamiento perfecto, por lo que en nuestro estudio se determinaron ambas en toda la población de estudio [14].

Encontramos un 4,7 % de pacientes de nuestra serie que presentan el genotipo CT de la variante rs1801158, y ningún paciente que presente el genotipo TT. La literatura describe un 9,3 % y 0,9 % para los genotipos CT y TT respectivamente.

Según nuestros resultados, podríamos concluir que nuestra población presenta un mayor porcentaje de pacientes con el genotipo CT de la variante rs1801160, y de genotipo GC de la variante rs75017182, pero menor frecuencia poblacional en la variante rs1801158, tanto en heterocigoto como homocigoto mutado.

Para las variantes rs1695 (*GSTP*), rs2228001 (*XPC*), rs3212986 (*ERCC1*), rs25487 (*XRCC1*), y rs11615 (*ERCC1*) implicados en el metabolismo y acción de los compuestos de platino, no se han encontrado diferencias significativas entre los genotipos de nuestra serie de pacientes, y los de la literatura, para población española [15].

En cuanto a los genes involucrados en respuesta y toxicidades del irinotecan, se han observado algunas discrepancias en la frecuencia poblacional española de las variantes del gen *UGT1A1*. La variante *1/*1 de nuestra serie presenta una frecuencia poblacional de 39,4 %, muy similar a la descrita para la población europea (40 %), mientras que la variante *28/*28 presenta una frecuencia de 19,7 %, superior a la descrita para la población española, que se describe del 9 %. El genotipo *1/*28 de nuestra serie se ha descrito en el 37,9 % de los pacientes estudiados, y la frecuencia descrita para la población española es de un 51 %. No se han descrito casos del genotipo *1/*36 en la literatura, sin embargo, en nuestra serie de pacientes hay un paciente que presenta este genotipo (3,0 %) [16].

Se identificaron las variantes rs1801158 y rs1801160 del gen *DPYD* con menores RAMs de tipo hematológico y GI grave, respectivamente. Otros autores han descrito resultados similares para la variante rs1801158 [17] si bien existe literatura en la que la variante rs1801160 se ha descrito como responsable de efectos adversos graves GI, difiriendo de nuestros resultados [18].

La menor supervivencia asociada a los genotipos CT de rs1801160 (p-valor: 0.006), el genotipo AT de la variante rs67376798 (p-valor<0.001), ambas para el gen *DPYD*, 1/*36 del gen *UGT1A1* (p-valor: 0.052), no ha sido descrita en la bibliografía consultada.

Con la creación del catálogo de pruebas genéticas de la cartera común de servicios del sistema nacional de salud, el Ministerio de Sanidad ha apostado por la oferta de servicios farmacogenéticos en los hospitales de todo el territorio nacional. Los genes y variantes señaladas para estudio en el caso de tratamiento del CCR, hasta el momento, son DPYD*2A, DPYD*13, *DPYD* c.2846A>T, *DPYD* c.1236G>A/HapB3; y *UGT1A1* [19]. Estudios como el realizado por nuestro grupo pretende ofrecer información que apoye la asociación entre variantes y fenotipos, con el objetivo de mejorar la asistencia sanitaria en la medicina personalizada.

Como podemos observar en nuestros resultados y conclusiones, es necesario hacer un estudio poblacional de los genes involucrados en las principales rutas metabólicas de fármacos empleados en el tratamiento del CCR para poder administrarlos adecuadamente.

### Limitaciones del estudio

Los resultados de este proyecto se encuentran dentro de la investigación de una Tesis Doctoral. Para la elaboración de esta tesis, se han recogido otras variables que pueden afectar a los resultados obtenidos, y que deben ser estudiados en futuras investigaciones.

Estos resultados preliminares deben ser contrastados y testados de nuevo en una cohorte de población más amplia, empleando estudios estadísticos más potentes, para validar su uso en la práctica clínica asistencial.
